# High Protein Binding and Cidal Activity against Penicillin-Resistant *S. pneumoniae:* A Cefditoren In Vitro Pharmacodynamic Simulation

**DOI:** 10.1371/journal.pone.0002717

**Published:** 2008-07-23

**Authors:** David Sevillano, Lorenzo Aguilar, Luis Alou, María-José Giménez, Natalia González, Martha Torrico, Fabio Cafini, Asunción Fenoll, Pilar Coronel, José Prieto

**Affiliations:** 1 Microbiology Department, School of Medicine, Universidad Complutense, Madrid, Spain; 2 Spanish National Reference Pneumococcal Laboratory, Instituto de Salud Carlos III, Madrid, Spain; 3 Scientific Department, Tedec-Meiji Farma SA, Madrid, Spain; Massachusetts General Hospital, United States of America

## Abstract

**Background:**

Although protein binding is a reversible phenomenon, it is assumed that antibacterial activity is exclusively exerted by the free (unbound) fraction of antibiotics.

**Methodology/Principal Findings:**

Activity of cefditoren, a highly protein bound 3^rd^ generation cephalosporin, over 24h after an oral 400 mg cefditoren-pivoxil bid regimen was studied against six *S. pneumoniae* strains (penicillin/cefditoren MICs; µg/ml): S1 (0.12/0.25), S2 (0.25/0.25), S3 and S4 (0.5/0.5), S5 (1/0.5) and S6 (4/0.5). A computerized pharmacodynamic simulation with media consisting in 75% human serum and 25% broth (mean albumin concentrations = 4.85±0.12 g/dL) was performed. Protein binding was measured. The cumulative percentage of a 24h-period that drug concentrations exceeded the MIC for total (T>MIC) and unbound concentrations (*f*T>MIC), expressed as percentage of the dosing interval, were determined. Protein binding was 87.1%. Bactericidal activity (≥99.9% initial inocula reduction) was obtained against strains S1 and S2 at 24h (T>MIC = 77.6%, *f*T>MIC = 23.7%). With T>MIC of 61.6% (*f*T>MIC = 1.7%), reductions against S3 and S4 ranged from 90% to 97% at 12h and 24h; against S5, reduction was 45.1% at 12h and up to 85.0% at 24h; and against S6, reduction was 91.8% at 12h, but due to regrowth of 52.9% at 24h. Cefditoren physiological concentrations exerted antibacterial activity against strains exhibiting MICs of 0.25 and 0.5 µg/ml under protein binding conditions similar to those in humans.

**Conclusions/Significance:**

The results of this study suggest that, from the pharmacodynamic perspective, the presence of physiological albumin concentrations may not preclude antipneumococcal activity of highly bound cephalosporins as cefditoren.

## Introduction

Antibiotics kill or inhibit the growth of bacteria by well known mechanisms of action. While in vitro antibiotics interact directly with bacteria without interference, in vivo (whether in natural or experimental infection) antibiotic and bacteria interactions are more complex due to the presence of serum proteins acting as an interface. Characterization of interactions between antibiotics and proteins is essential in the assessment of pharmacodynamic implications on antibacterial activity. While some serum proteins, as gammaglobulins and complement enhance the antipneumococcal activity of β-lactams as 3^rd^ generation cephalosporins [1], [2], others as albumin produce limitations in antibacterial activity in highly protein bound agents. However, although it is generally accepted that only the unbound fraction of the antibiotic is active in vitro (and presumably in vivo), the quick reversibility of the protein drug binding indicates that the presumed limitations of antimicrobial activity can be far from absolute, also for highly protein bound antibiotics [3].

Results of previous studies focusing on the effects of protein binding on antimicrobial activity against gram-positive microorganisms ranged from impairment to delay [4], [5] or even increase of activity [6]. As with other antibiotics [7], attempts to interpret cefditoren pharmacodynamics using reported percentages of protein binding can be inappropriate [8]. In the case of β-lactams and *Streptococcus pneumoniae*, 33% time above the minimum inhibitory concentration (T>MIC) has been previously used as a bacteriostatic endpoint [9] and 40% T>MIC as the predictive value for clinical cure in humans [10]. The pharmacodynamic parameter predicting β-lactam efficacy (T>MIC) is usually based on free-drug concentrations extrapolated from total drug concentrations by considering the protein binding, thus estimating antibiotic activity under the most stringent conditions.

Cefditoren is a third generation oral cephalosporin exhibiting in vitro higher intrinsic activity than cefotaxime against *S. pneumoniae* [11], [12], a peak serum concentration of 4.2 µg/ml after a 400 mg single dose, a protein binding rate of 88% [13], and clinical efficacy in respiratory tract infections [14], [15].

Computerized pharmacodynamic devices allow the study of antibacterial activity along the dosing interval of simulated serum concentrations, after standard dosing regimens, against specific strains. Conclusions on antibiotic therapeutic activity (eradication and subsequent clinical cure), and its pharmacodynamic prediction, against these specific strains (with high MIC to the target antibiotic) cannot be drawn from clinical trials due to the scarce number of patients infected with these strains. The aim of this study was to evaluate the bactericidal activity over 24h of simulated serum concentrations of cefditoren obtained after an oral 400 mg cefditoren-pivoxil bid regimen against penicillin non-susceptible *S. pneumoniae* in an in vitro pharmacodynamic simulation with human serum containing albumin physiological concentrations.

## Results

**Figure 1:**
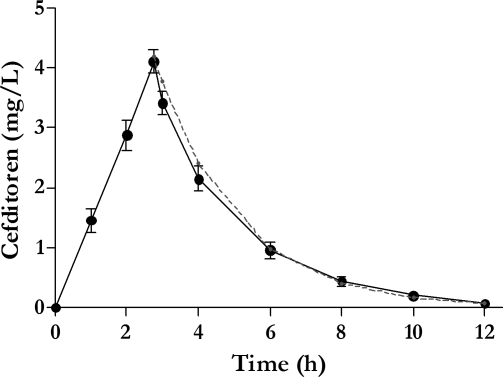
Cefditoren concentrations: Mean±SD target (grey dotted line) and experimental (black line) concentrations in the simulations.

Figure 1 shows target and experimental concentrations of cefditoren in the simulation process. Experimental pharmacokinetic parameters obtained were: Cmax = 4.1±0.2 µg/ml, Tmax = 2.8±0.0 h,t1/2 = 1.6±0.1 h, and AUCall = 14.3±0.8 µg/ml×h. Mean albumin concentration was 4.9±0.1 g/dl at 0h, 4.8±0.2 g/dl at 12h and 4.8±0.1 g/dl at 24h. pH ranged from 7.4 to 7.8. Protein binding of cefditoren in the media used in the simulation (75% non-heat inactivated human serum and 25% Mueller-Hinton broth supplemented with 5% lysed sheep blood; HS-SMH) was 85.7±1.8% for 4 µg/ml, 88.2±0.6% for 0.5 µg/ml, and 87.5±1.2% for 0.125 µg/ml (mean 87.1±1.3%; coefficient of variation = 0.0149).

Susceptibilities of strains to penicillin and cefditoren and serotypes are shown in Table 1. The same MIC values were found prior and after the simulations carried out.

**Table 1:** Mean reduction of initial inocula: Time to obtain 90%, 99% and 99.9% reductions (T_90%_, T_99%_ and T_99.9%_), maximal effect (maximal reduction in percentage within the dosing interval: 0-12h) and percentage of reduction at 12h and 24h.

Strain (MIC_PEN_)	MIC_CDN_	T>MIC_CDN_	*f*T>MIC_CDN_	Initial inocula	T_90%_	T_99%_	T_99.9%_	Maximal effect	Percentage of reduction of initial inocula	Strain (MIC_PEN_)
									**12h**	**24h**
**S1**_(0.12)_	0.25	77.6	23.7	5.5×10^7^	3h	4h	6h	>99.9	99.6	>99.9
**S2**_(0.25)_	0.25	77.6	23.7	2.5×10^7^	3h	4h	6h	>99.9	98.1	>99.9
**S3**_(0.5)_	0.5	61.6	1.7	5.5×10^7^	2h	4h	-	99.8	94.2	96.8
**S4**_(0.5)_	0.5	61.6	1.7	4.8×10^7^	6h	-	-	94.3	90.7	96.6
**S5**_(1)_	0.5	61.6	1.7	5.5×10^7^	6h	-	-	94.0	45.1	85.0
**S6**_(4)_	0.5	61.6	1.7	1.9×10^7^	6h	-	-	98.4	91.8	52.9

PEN = penicillin, CDN = cefditoren.

T>MIC: % dosing interval with total cefditoren concentrations over MIC. *f*T>MIC: % dosing interval with free-cefditoren concentrations over MIC.

**Figure 2:**
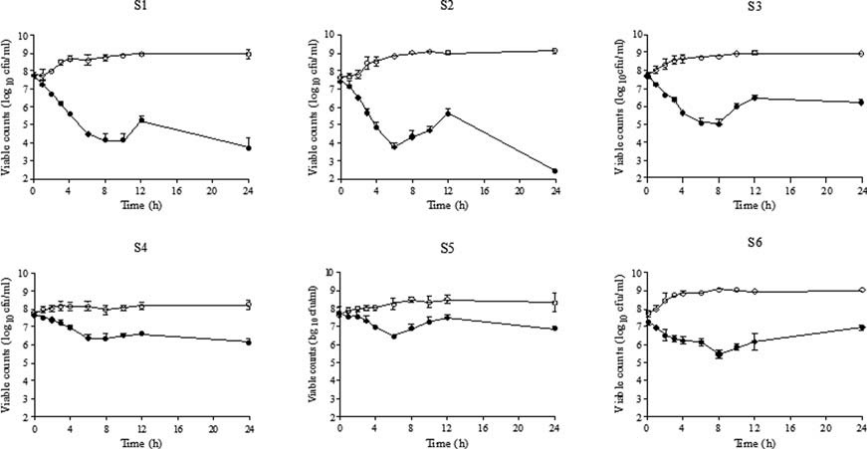
Cefditoren antibacterial activity over time: Mean bacterial counts over 24h in antibiotic-free (open circles) and cefditoren (black circles) simulations.

**Figure 3:**
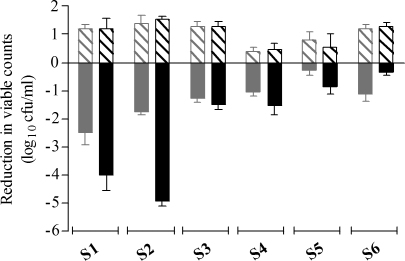
Mean changes in initial inocula at the end of dosing intervals: Decrease in log_10_ cfu/ml initial inocula in antibiotic simulations at 12h (solid grey) and 24h (solid black); or increase in log_10_ cfu/ml initial inocula in antibiotic-free simulations at 12h (grey diagonal stripes) and at 24h (black diagonal stripes).

Figure 2 shows per strain mean bacterial counts over 24h, and Figure 3, mean changes in log_10_ cfu/ml at 12h and 24h in antibiotic-free and cefditoren simulations. All strains but S6 exhibited higher reduction in viable counts (log_10_ cfu/ml) at 24h than at 12h.

Table 1 shows experimental cumulative percentage of a 24 h period that drug concentrations exceeded the MIC for total (T>MIC) and unbound concentrations (*f*T>MIC) expressed as percentage of the dosing interval, initial inocula, times to obtain 90% (T_90%_), 99% (T_99%_) and 99.9% (T_99.9%_) reductions in initial inocula, maximal reduction within the dosing interval (i.e, 12h) and percentages of reduction at 12h and 24h. T_90%_ was ≤6h for all strains regardless the 0.25 or 0.5 µg/ml value of cefditoren MIC, T_99%_ was 4h for strains S1, S2 and S3 (MICs of 0.25, 0.25 and 0.5 µg/ml, respectively), and T_99.9%_ was 6h for the two strains exhibiting cefditoren MIC of 0.25 µg/ml. The maximal effect (maximal reduction in initial inocula) was ≥94% for all strains, being ≥99.9% for strains exhibiting cefditoren MIC of 0.25 µg/ml.

Cefditoren, with a T>MIC of 77.6% and *f*T>MIC of 23.7% maintained at 24h its bactericidal activity (≥99.9% reduction) against strains with cefditoren MIC = 0.25 µg/ml (S1 and S2). Against strains inhibited by 0.5 µg/ml cefditoren, with T>MIC of 61.6% and *f*T>MIC of 1.7%, cefditoren reduced in 90–97% the initial inocula of S3 and S4 (penicillin MIC of 0.5 µg/ml), and in 45.1% (at 12h) and up to 85.0% (at 24h) the initial inocula of S5 (penicillin MIC of 1 µg/ml). Against strain S6 (penicillin MIC of 4 µg/ml), cefditoren produced a reduction in initial inocula of 91.8% at 12h but, due to the subsequent regrowth, of 52.9% at 24h.

## Discussion

The dominant pharmacodynamic index determining effects of β-lactams against *S. pneumoniae* is the time that drug concentrations remains over the MIC, and determining the size of this pharmacodynamic index for predicting optimal antibacterial activity is critical and helps to determine dosing regimens, breakpoints, and hence relevant clinical resistance [16]. The magnitude of the parameter predicting the maximal effect in vivo intuitively depends on inoculum, strain, protein binding, and immune status.

To our knowledge this is the first study experimentally analysing, from a dynamic point of view, the impact of protein binding on the antipneumococcal activity of an antibiotic, by using an in vitro pharmacodynamic system with a matrix mimicking the physiological situation. This matrix consisted in 25% broth and 75% human serum, and contained 4.85 g/dl human albumin, a value in the upper limit of the normal range in humans (3.5–5 g/dl), with a constant physiological pH value of 7.4–7.8. With respect to bacterial growth, this media in simulations without antibiotics supported adequate growth of the *S. pneumoniae* strains along the simulation period. Some key differences between in vivo and in vitro pharmacokinetic models are the inoculum (bacterial load), usually higher in in vitro models [16], and the absence of immune system in in vitro models. However it can be postulated that the absence of immune system is compensated by the microorganism clearance from one-compartment systems. In this study we used a high inoculum to more closely mimic human infection, and a one-compartment model with a favourable ratio of multiplication rate/clearance of the microorganism from the system with the media used (controls in Figure 2).

With respect to the antibiotic, experimental protein binding of cefditoren in the matrix was 87.1%, a rate comparable with that described in humans (88%) in the literature [13]. Although pH does not appear to be an issue in cephalosporin protein binding [17], in this study the pH was maintained at within physiological values of 7.4–7.8.

With respect to the strains used, although there are not CLSI defined breakpoints for cefditoren [18], according to FDA proposed breakpoints (≤0.125 µg/ml for susceptibility and ≥0.50 µg/ml for resistance [19]) two strains should be considered as intermediate (cefditoren MIC of 0.25 µg/ml) and four resistant (cefditoren MIC of 0.5 µg/ml).

Despite the experimentally confirmed high rate of cefditoren protein binding, in the present simulations cefditoren decreased >99.9% initial inocula (3 log_10_ reduction; bactericidal activity) from 6h on of strains exhibiting cefditoren MIC of 0.25 µg/ml. These reductions were obtained with T>MIC of 77.6% (a value higher than the 40% predictive value for clinical cure in humans [10]), but *f*T>MIC of 23.7%, a value lower than the 33% classically considered for bacteriostatic effect [9]. With respect to strains exhibiting cefditoren MIC of 0.5 µg/ml, a maximal effect of initial inocula reduction ranging from 94.0 to 99.8% (higher than inhibitory effect) was obtained with a T>MIC of 61.6%, but *f* T>MIC of only 1.7%. The results of this study suggest that, from the pharmacodynamic perspective, the presence of physiological albumin concentrations does not preclude activity of cefditoren physiological concentrations over the dosing interval against the high inocula of the strains tested.

 All strains, but strain S6, exhibited initial inocula reductions higher at 24h than at 12h, reaching at the end of the experiment bactericidal activity in the case of the strains with 0.25 µg/ml and initial inocula reductions from 85.0% to 96.8% in the case of the three penicillin intermediate resistant strains exhibiting cefditoren MIC of 0.5 µg/ml. This suggests that against these latter strains additional doses in the model (simulating bid regimens for 48h or 72h) may result in higher effect, probably reaching the bactericidal end-point.

The situation is completely different with S6, the strain exhibiting cefditoren MIC of 0.5 µg/ml that was penicillin-resistant (MIC = 4 µg/ml). This strain, as the others, exhibited the same MIC values pre- and post-simulation, but can be considered phenotypically tolerant to cefditoren physiological concentrations because of the regrowth that occurred from 12h (91.8% initial inocula reduction) to 24h (52.9% reduction), thus not expecting bactericidal activity with bid regimens for 48h or 72h). This regrowth pattern was observed with amoxicillin/clavulanic acid in highly penicillin-resistant strains [20], and is attributable to defective lysis that is more prevalent in penicillin-resistant pneumococci [21]. Tolerance is attributable to changes in autolysin activity control rather than to survival after bactericidal doses [21].

 “Susceptibility” is classically defined as pathogen likely inhibition by blood concentrations, and “resistance” as pathogen not likely inhibited by blood concentrations usually achieved [18], [19]. If the results obtained in this study are confirmed with different penicillin-resistant serotypes, and provided there is clinical evidence of eradication, strains exhibiting 0.25 and 0.5 µg/ml cefditoren MICs may not be considered as non-susceptible because physiological cefditoren concentrations along 24h (after a 400 mg bid regimen), in a media where its protein binding was 87.1%, were bactericidal in the first case and in the second produced not simple inhibition and were more than bacteriostatic.

## Materials and Methods

### Strains

Six penicillin non-susceptible *S. pneumoniae* isolates of different serotypes from the Spanish Pneumococcal Reference Laboratory (Instituto de Salud Carlos III) were used throughout this study. Strains were chosen on the basis of cefditoren MIC values matching cefditoren MIC_50_ and MIC_90_ values for penicillin non-susceptible strains in previous studies [11], [12], [22]: two strains with cefditoren MIC of 0.25 µg/ml and four strains with cefditoren MIC of 0.5 µg/ml. Serotypes and penicillin MIC for the strains were: S1 (serotype 15A; 0.12 µg/ml), S2 (serotype 6B; 0.25 µg/ml), S3 (serotype 19F; 0.5 µg/ml), S4 (serotype 6A; 0.5 µg/ml), S5 (Non-typable; 1 µg/ml), and S6 (serotype 9V; 4 µg/ml).

Prior and after the simulation process, MICs were determined in quintuplicate using broth microdilution method following CLSI recommendations [23], and modal values were considered.

### Media

Culture media used in all pharmacokinetic/pharmacodynamic (PK/PD) experiments consisted in a matrix of 75% non-heat inactivated human serum (batch 01111695; Lonza Group Ltd, Basel, Switzerland) and 25% Mueller-Hinton broth (Difco laboratories, Detroit, Mi.) supplemented with 5% lysed sheep blood (Difco laboratories) (HS-SMH), with a final pH of 7.7. Previous experiments were performed to confirm that HS-SMH was able to sustain the growth of *S. pneumoniae*.

Broth and agar Mueller-Hinton supplemented with 5% lysed sheep blood were used for susceptibility tests and recovery of viable bacteria in PK/PD experiments.

### In vitro kinetic model

A one-compartment dynamic model was used to expose bacteria to changing study drug concentrations [24]. The system is represented by three connected flask: (i) a fresh media reservoir containing HS-SMH (ii) a central flask with multiple ports for addition and removal of broth, delivery of the antibiotic, and collection of bacterial and antibiotic samples, containing media plus bacterial culture, and (iii) a waste compartment. A computer-controlled syringe pump (402 Dilutor Dispenser; Gilson S.A, Villiers-le-Bel, France) infused the drug into the central flask until the Cmax was reached. The exponential decay of antibiotic concentrations was achieved by a continuous dilution-elimination process using computerized peristaltic pumps (Masterflex, Cole-Parmer Instrument Co., Chicago, Ill.) at a rate of 0.45 ml/min, set to simulate the half-live of cefditoren [25], [26] in humans. The same flow rate for media removal allowed a constant volume all over the simulation. The entire model was maintained at 37°C during the simulation process with magnetic stir bars in the media to allow for continuous mixing. Growth controls experiments, were performed in the same conditions without cefditoren.

### Kinetic simulations

The pharmacokinetic profile in serum after oral cefditoren-pivoxil 400 mg bid [25], [26], [27] was simulated over 24h. Target pharmacokinetic parameters, based on mean values reported in humans [25], [26], [27], were Cmax = 4.09 µg/ml, Tmax = 2.67h, t1/2 = 1.53h, and AUCall = 14.28 µg/ml×h.

### Experimental protein binding and albumin concentrations

Cefditoren protein binding in HS-SMH was measured by an ultrafiltration method described by Craig and Suh [17]. Cefditoren was added to HS-SMH at concentrations of 4 (concentration similar to Cmax), 0.5, and 0.125 µg/ml. A 1-ml aliquot of each sample was transferred to a centrifugal system device (Centrifree®, Amicon Bioseparations, Millipore, Tullagreen, Ireland) and was centrifuged at 1,200 g for 15 min at room temperature in a Beckman fixed-angle rotor centrifuge (model L8-55, Beckman Instrument, Fullerton, CA, USA). Cefditoren concentrations in pre-filtered samples and in ultrafiltrates recovered in the polyethylene filtrate cups, were measured by bioassay (see below). Percentage of cefditoren bound to proteins in HS-SMH was calculated using the expression:
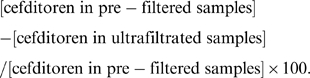



Protein binding studies were conducted in triplicate for each drug concentration examined.

In each simulation, pH and albumin concentrations were measured at 0, 12 and 24h by the bromcresol green method [28].

### Pharmacokinetic analysis

Simulated cefditoren concentrations were measured in samples (0.5 ml) taken at 1, 2, 2.67 (target Tmax), 3, 4, 6, 8, 10, 12 and 24h in triplicate by bioassay [29] using *Morganella morganii* ATCC 8076H as indicator organism [30]. Plates were inoculated with an even lawn of indicator organism and incubated for 18 to 24h at 37°C. Standards and dilutions were prepared in HS-SMH and in saline as protein-free control solution for ultrafiltrated samples (lineal concentrations from 0.015 to 4 µg/ml (r>0.99); limit of detection = 0.015 µg/ml). Intra-day and inter-day coefficient of variations were 5.83% and 3.16% for a concentration of 0.75 µg/ml in HS-SMH and 2.83% and 3.55% in saline, respectively.

Antimicrobial concentrations were analyzed by a non-compartmental approach using WinNonlin 5.2 Professional program (Pharsight, Mountainview, Ca.). Cmax and Tmax were obtained directly from observed data and the area under the concentration-time curve (AUC_0–12_) was calculated by the linear-log trapezoidal rule. Both total and free-drug concentrations (according to protein binding in HS-SMH) were considered throughout the study. The cumulative percentage of a 24 h period that drug concentrations exceeded the MIC for total (T>MIC) and unbound concentrations (*f*T>MIC) expressed as percentage of the dosing interval, were calculated by a non-compartmental approach for pharmacodynamic data using the model 220 of WinNonlin program.

### Measurement of antibacterial effect

Colonies from an overnight culture were allowed to grow in Mueller-Hinton broth supplemented with 5% lysed sheep blood to a density ranging from 1×10^8^ to 2×10^8^ cfu/ml, as measured by a UV- spectrophotometer (Hitachi U-1100). A 6 ml aliquot of this bacterial suspension were added into the central flask containing 60 ml of a 37°C atempered HS-SMH one hour before the simulation process to allow the microorganism adaptation to the medium. Initial inocula in all simulations ranged between 2×10^7^–6×10^7^ cfu/ml. Samples (0.5 ml) were collected at 0, 1, 2, 3, 4, 6, 8, 10, 12 and 24h, and serially diluted in 0.9% sodium chloride. At least four dilutions of each sample were spread (20 µl) onto Mueller-Hinton agar supplemented with 5% sheep blood and overnight incubated for colony counting. The limit of detection was 50 cfu/ml. Each experiment was performed in triplicate. Changes in viable counts between time 0 and the different timepoints were calculated and expressed as percentage of reduction. Times to obtain 90% (T_90%_), 99% (T_99%_) and 99.9% (T_99.9%_) reductions were determined.
